# Children with sickle cell disease and severe COVID‐19 presenting single nucleotide polymorphisms in innate immune response genes – A case report

**DOI:** 10.1002/jha2.325

**Published:** 2021-11-24

**Authors:** Natália Lima Pessoa, Lilian Martins Oliveira Diniz, Adriana de Souza Andrade, Erna Geessien Kroon, Aline Almeida Bentes, Marco Antônio Campos

**Affiliations:** ^1^ Laboratório de Vírus Departamento de Microbiologia Instituto de Ciências Biológicas Universidade Federal de Minas Gerais Belo Horizonte Brazil; ^2^ Imunologia de Doenças Virais, Instituto René Rachou Fundação Oswaldo Cruz Belo Horizonte Brazil; ^3^ Departamento de Pediatria, Faculdade de Medicina Universidade Federal de Minas Gerais Belo Horizonte Brazil; ^4^ Hospital João Paulo II Fundação Hospitalar do Estado de Minas Gerais Belo Horizonte Brazil

**Keywords:** case report, COVID‐19, innate immunity, sickle cell disease, single nucleotide polymorphisms, TIRAP, TLR‐7

## Abstract

Here we report three clinical cases of children with sickle cell disease (SCD) and severe COVID‐19 who evolved with complications during hospitalization or after discharge. They present single nucleotide polymorphisms in *tlr‐7* and *tirap* genes, identified from 37 patients under 16 years old hospitalized from September 2020 to May 2021 in the Hospital João Paulo II, Belo Horizonte, Brazil. They presented significant complications of SCD as acute chest syndrome, splenic sequestration, and pain crisis during hospitalization or up to 2 months after SARS‐CoV‐2 infection. They all required transfusion of concentrated red blood cells and hospitalization in a reference hospital to care for children with SCD.

Sickle cell disease (SCD) is a clinical expression of homozygosity for hemoglobin S characterized by chronic hemolytic anemia. Worldwide it is estimated that 3.2 million people are living with SCD. SCD patients may develop acute chest syndrome, pulmonary embolism, and stroke [1]. The innate immune system is activated in SCD with a high expression of toll‐like receptor 4 (TLR‐4) [2], which depends on Tir domain‐containing adaptor protein to be functional. Additionally, there is an involvement of innate immune cells, including monocytes, neutrophils, platelets, and mast cells, promoting inflammation, adhesion, and pain [3].

SARS‐CoV‐2 is a single‐stranded RNA betacoronavirus responsible for the COVID‐19 pandemic. Studies in children infected with SARS‐CoV‐2 in developed countries have detected low mortality. However, a recent study found that children and adolescents who had a previous disease, such as SCD, had a 2.96 risk of evolving to death [4].

The host's innate immune system is responsible for protecting against microorganisms [5, 6], including viruses [7, 8, 9], and there is a recognition of pathogen‐associated molecular patterns through their pattern recognition receptors [5, 6, 7, 8, 9, 10]. TLR‐7 is a pattern recognition receptor that recognizes single‐stranded RNA [10]. Most TLR signaling initiates with the activation of adaptor protein MyD88, which is recruited to the Tir domain present in the cytosolic tail of all TLRs. In response to natural activators of innate immunity, the sorting adaptor Tir domain‐containing adaptor protein regulates TLR signaling from the plasma membrane and endosomes [11]. Single nucleotide polymorphisms (SNPs) in innate immune system genes can negatively influence the immune response to infectious diseases [12], as exemplified by rs179008 (*tlr‐7*) [12] and rs8177374 (*tirap*) [13]. Chromosomal X *tlr‐7 l*oss‐of‐function variants were identified in four young men with severe COVID‐19 [14].

Here we report three clinical cases of children with SCD and severe COVID‐19 who evolved with complications during hospitalization or after discharge (Table 1). These patients with SCD, SARS‐CoV‐2 positive, with SNPs present in *tlr‐7* and *tirap* genes, were identified over 9 months (from September 2020 to May 2021) in the Hospital João Paulo II, Belo Horizonte, Brazil, from 37 cases of severe COVID‐19 studied.

**TABLE 1:** Laboratory results, SARS‐CoV‐2 test, and single nucleotide polymorphism genotype and phenotype

**Tests (units) demographic /patient**	**1**	**2**	**3**	**Reference range**
Age (years, months)	15	1, 5	6	–
Sex	Feminine	Masculine	Feminine	–
Oxygen saturation at hospital admission	90%	98%	80%	94%–100%
Clinical manifestation	Acute chest syndrome	Flu syndrome	Viral pneumonia	–
Chest tomography	Diffuse interstitial infiltrate. Bronchial‐vascular cluster at the base of the right lung.	Not performed	Interstitial infiltrate of the posterior and inferior segment of the right hemithorax; bilateral bronchial thickening.	–
qRT SARS‐CoV‐2	P	P	P	N
SARS‐CoV‐2 Antigen	ND	ND	P	N
Hemoglobin (g/fdl)	6.3	7.7	5.5	11.5–13.5
Reticulocytes (%)	0.1	7.8	14.2	1–1.5
Leukocytes (cells × 10^3^/mm^3^)	11.8	3.9	21.9	5–14.5
Neutrophil (cells × 10^3^/mm^3^)	7.0	1.5	16.4	1.5–8.0
Lymphocyte (cells × 10^3^/mm^3^)	3.2	2.2	2.8	1.5–6.5
Platelets (cells/mm^3^ × 10^3^)	418	87	195	150–400
C‐reactive protein (mg/L)	49.6	6.0	85.9	<12
Blood culture	N	N	N	N
TLR‐7 T/T (A/A)	HT	NH	NH	–
TIRAP C/C (T/T)	NH	HT	HT	–

Abbreviations: HT, heterozygote; N, negative; ND, not done; NH, normal homozygote; P, positive; TIRAP, toll‐interleukin 1 receptor domain‐containing adapter protein; TLR‐7, toll‐like receptor 7.

The identified SNPs rs179008 in the *tlr‐7* gene and rs8177374 in the *tirap* gene were tested using the methods described before [15]. A base change (A > T) was detected on rs179008 (*tlr‐7*) SNP, causing an amino acid alteration (Q to L), and additionally, there was a base alteration (C > T) with a change of S to L in SNP rs8177374 (*tirap*).

The procedures were in accord with the Committee on Human Experimentation from Instituto René Rachou, Fundação Oswaldo Cruz [CAAE 37207920.6.0000.5091] and with the Helsinki Declaration (1964, amended most recently in 2008) of the World Medical Association and the patient's responsible written consent was obtained.

The first patient, a 15‐year‐old female, was admitted to the hospital with acute chest syndrome 1 week after the onset of SARS‐CoV‐2 symptoms. She had severe pain in the sternum and bilateral chest, ventilatory dependent. Nasal cannula oxygen therapy 2l/min for 8 days, morphine for pain control for 5 days was required. She was medicated with antibiotics for 10 days, with 40 mg/day enoxaparin and inhaled salbutamol throughout her hospital stay. On the third day of hospitalization, a real‐time reverse transcription‐polymerase chain reaction (qRT ‐PCR) for SARS‐CoV‐2 from the collected nasopharyngeal swab was positive. On the fifth day of hospitalization, she evolved with significant pallor associated with splenomegaly (palpable spleen 6 cm from the right costal margin). A total blood count showed a drop in hemoglobin from 8.5 to 6.3 mg/dl, with no reticulocyte increase (0.1%). She received red blood cell transfusions, with gradual improvement, and oxygen therapy was discontinued 3 days later. She was discharged after 11 days of hospitalization. The SNP rs179008 in the *tlr‐7* gene was identified in this patient.

The second patient, a child aged 1 year and 5 months, was admitted to the hospital after 3 days of coughing, fever, and sore throat. He evolved with diarrhea and pallor, with a drop in hemoglobin from 8.5 to 7.7 mg/dl. A nasopharyngeal swab for SARS‐CoV‐2 was collected on the fourth day of the onset of symptoms which qRT‐PCR was positive. He was discharged after 3 days. However, 2 months later, he was admitted to the intensive care unit in another hospital due to splenic sequestration and received a blood transfusion. The SNP rs8177374 in the *tirap* gene was identified in this patient.

The third patient, a 6‐year‐old child, was admitted to the hospital one day after the onset of symptoms as cough, fever, and dyspnea. She was admitted to the emergency room due to tachypnea and oxygen saturation of 80%, and oxygen was administered through a non‐rebreathing mask. Chest tomography showed an image of interstitial involvement in the posterior and inferior segment of the right thorax and bilateral bronchial thickening. A nasopharyngeal swab for SARS‐CoV‐2 antigen was collected on the first day of the onset of symptoms, and qRT‐PCR was positive. Two days after discharge, she returned to the hospital with an intense pain crisis associated with oliguria, decreased oxygen saturation, and fever. Intravenous hydration, fixed morphine, ceftriaxone, and oxygen through nasal cannula were prescribed. Three days later, she developed paleness and a drop in hemoglobin from 7.5 to 5.5 mg/dl. She received a red blood cell transfusion with progressive improvement, being discharged after a week with reasonable pain control. The SNP rs8177374 in the *tirap* gene was identified in this patient.

The children reported here presented significant complications of SCD as acute chest syndrome, splenic sequestration, and pain crisis during hospitalization or up to 2 months after SARS‐CoV‐2 infection. They all required transfusion of concentrated red blood cells and hospitalization in a reference hospital to care for children with SCD. We studied 37 cases of severe COVID‐19, with three SCD patients, corresponding to 8.1% of all studied cases. All the SCD patients present SNPs, which draws our attention, why we wrote this report. We want to stimulate further studies to draw attention to the possible impact in children with SCD, COVID‐19, and SNPs in the TLR pathway.

## AUTHOR CONTRIBUTIONS

Natália Lima Pessoa, Aline Almeida Bentes, Erna Geessien Kroon, and Marco Antônio Campos conceived and designed the experiments. Natália Lima Pessoa performed the experiments. Natália Lima Pessoa, Adriana de Souza Andrade, Erna Geessien Kroon, Aline Almeida Bentes, and Marco Antônio Campos analyzed the data. Marco Antônio Campos and Erna Geessien Kroon contributed reagents, materials, and analysis tools. Aline Almeida Bentes and Lilian Martins Oliveira Diniz did attendance and medical assistance to and blood collecting from the children. Natália Lima Pessoa, Adriana de Souza Andrade, Erna Geessien Kroon, Aline Almeida Bentes, and Marco Antônio Campos wrote the paper. All authors read and approved the final manuscript.

## References

[1] Sundd P , Gladwin MT , Novelli EM . Pathophysiology of sickle cell disease. Annu Rev Pathol. 2019;14:263–92 30332562 10.1146/annurev-pathmechdis-012418-012838 PMC7053558

[2] Kato GJ , Piel FB , Reid CD , Gaston MH , Ohene‐Frempong K , Krishnamurti L , et al. Sickle cell disease. Nat Rev Dis Primers. 2018;4:18010. 10.1038/nrdp.2018.10 29542687

[3] Allali S , Maciel TT , Hermine O , de Montalembert M . Innate immune cells, major protagonists of sickle cell disease pathophysiology. Haematologica 2020;105(2):273–83. 31919091 10.3324/haematol.2019.229989 PMC7012475

[4] Oliveira EA , Colosimo EA , Simões E , Silva AC , Mak RH , Martelli DB , et al. Clinical characteristics and risk factors for death among hospitalised children and adolescents with COVID‐19 in Brazil: an analysis of a nationwide database. Lancet Child Adolesc Health. 2021;5(8):559–68. 34119027 10.1016/S2352-4642(21)00134-6 PMC8192298

[5] Campos MA , Almeida IC , Takeuchi O , Akira S , Paganini E , Procopio DO , et al. Activation of Toll‐like receptor‐2 by glycosylphosphatidylinositol anchors from a protozoan parasite. J Immunol. 2001;167:416–423. 11418678 10.4049/jimmunol.167.1.416

[6] Gazzinelli RT , Ropert C , Campos MA . Role of the Toll/Interleukin‐1 receptor signaling pathway in host resistance and pathogenesis during infection with protozoan parasites. Immunol Rev. 2004;201:9–25. 15361229 10.1111/j.0105-2896.2004.00174.x

[7] Mansur DS , Kroon EG , Nogueira M , Arantes RME , Rodrigues SCO , Akira S , et al. Lethal encephalitis in myeloid differentiation factor 88‐deficient mice infected with Herpes simplex virus 1. Am J Pathol. 2005;166:1419–26. 15855642 10.1016/S0002-9440(10)62359-0 PMC1606396

[8] Lima GK , Zolini GP , Mansur DS , Lima BHF , Wischhoff U , Astigarraga RG , et al. Toll‐like receptor (TLR) 2 and TLR9 expressed in trigeminal ganglia are critical to viral control during herpes simplex virus 1 infection. Am J Pathol. 2010;177:2433–45. 20864677 10.2353/ajpath.2010.100121 PMC2966801

[9] Zolini GP , Lima GK , Lucinda N , Silva MG , Dias MF , Pessoa NL , et al. Defense against HSV‐1 in a murine model is mediated by iNOS and orchestrated by the activation of TLR2 and TLR9 in trigeminal ganglia. J Neuroinflammation. 2014;11:20. 24479442 10.1186/1742-2094-11-20 PMC3922087

[10] Kawai T , Akira S . Innate immune recognition of viral infection. Nat Immunol. 2006;7:131–7. 16424890 10.1038/ni1303

[11] Bonham KS , Orzalli MH , Hayashi K , Wolf AI , Glanemann C , Weninger W , et al. A promiscuous lipid‐binding protein diversifies the subcellular sites of toll‐like receptor signal transduction. Cell 2014;156(4):705–16. 24529375 10.1016/j.cell.2014.01.019 PMC3951743

[12] Mukherjee S , Huda S , Babu SPS . Toll‐like receptor polymorphism in host immune response to infectious diseases: a review. Scand J Immunol. 2019;90:e12771. 31054156 10.1111/sji.12771

[13] Alagarasu K , Honap T , Damle IM , Mulay AP , Shah PS , Cecilia D . Polymorphisms in the oligoadenylate synthetase gene cluster and its association with clinical outcomes of dengue virus infection. Infect Genet Evol. 2013;14:390–5. 23337612 10.1016/j.meegid.2012.12.021

[14] van der Made CI , Simons A , Schuurs‐Hoeijmakers J , van den Heuvel G , Mantere T , Kersten S , et al. Presence of genetic variants among young men with severe COVID‐19. JAMA 2020;324:663–73. 32706371 10.1001/jama.2020.13719 PMC7382021

[15] Pessoa NL , Bentes AA , de Carvalho AL , de Souza Silva TB , Alves PA , de Sousa Reis EV , et al. Case report: hepatitis in a child infected with SARS‐CoV‐2 presenting toll‐like receptor 7 Gln11Leu single nucleotide polymorphism. Virol J. 2021;18:180 34482844 10.1186/s12985-021-01656-3 PMC8418785

